# Supplementary figures and images for: Trait Mapping of Phenolic Acids in an Interspecific (Vaccinium corymbosum var. caesariense × V. darrowii) Diploid Blueberry Population
Source: Plants (Basel). 2023 Mar 16;12(6):1346. doi: 10.3390/plants12061346 (PMC10057043; doi:10.3390/plants12061346)

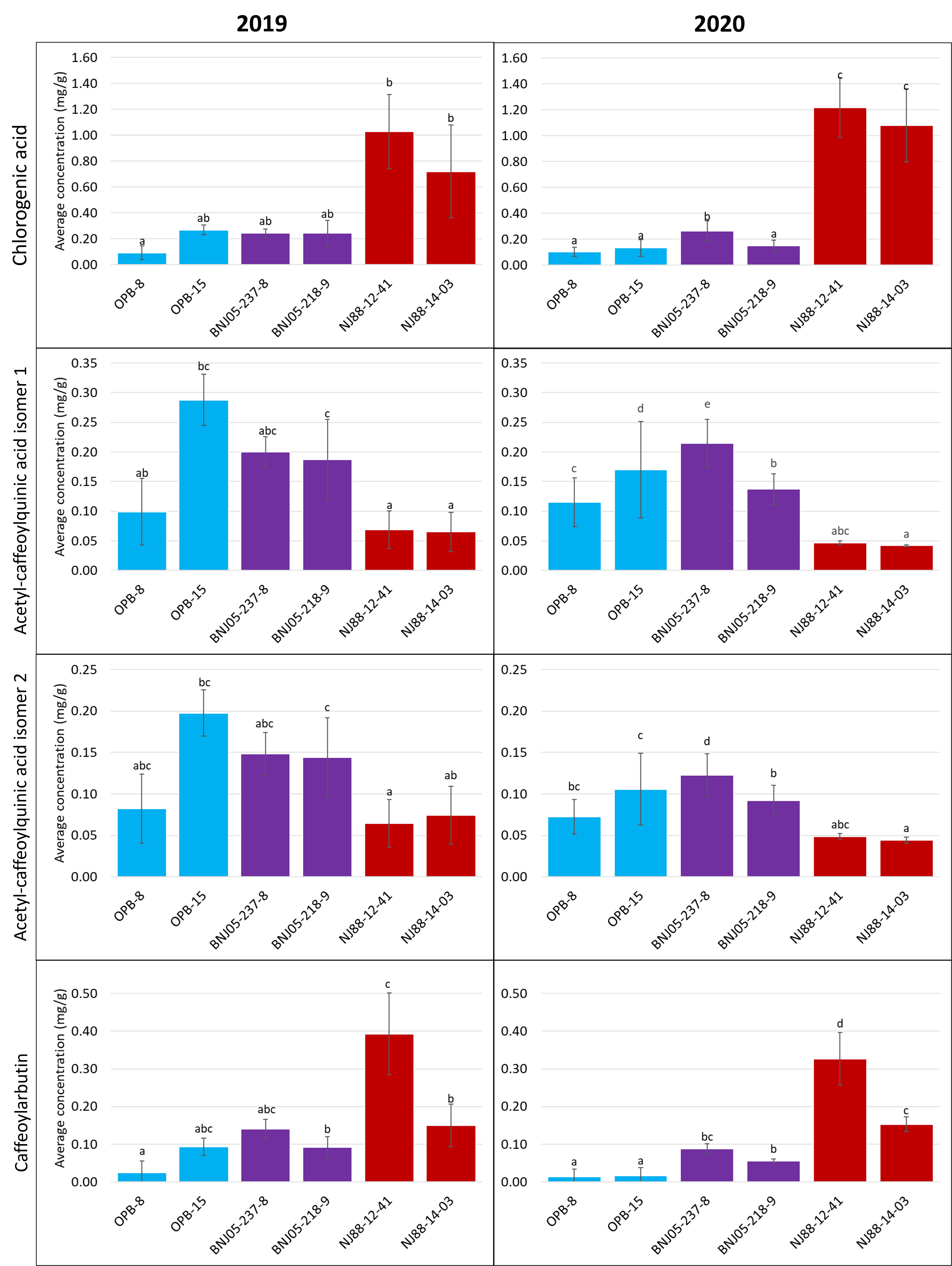

Supplement: Supplementary file 1 [file plants-12-01346-s001.zip › Figure_S1.png]

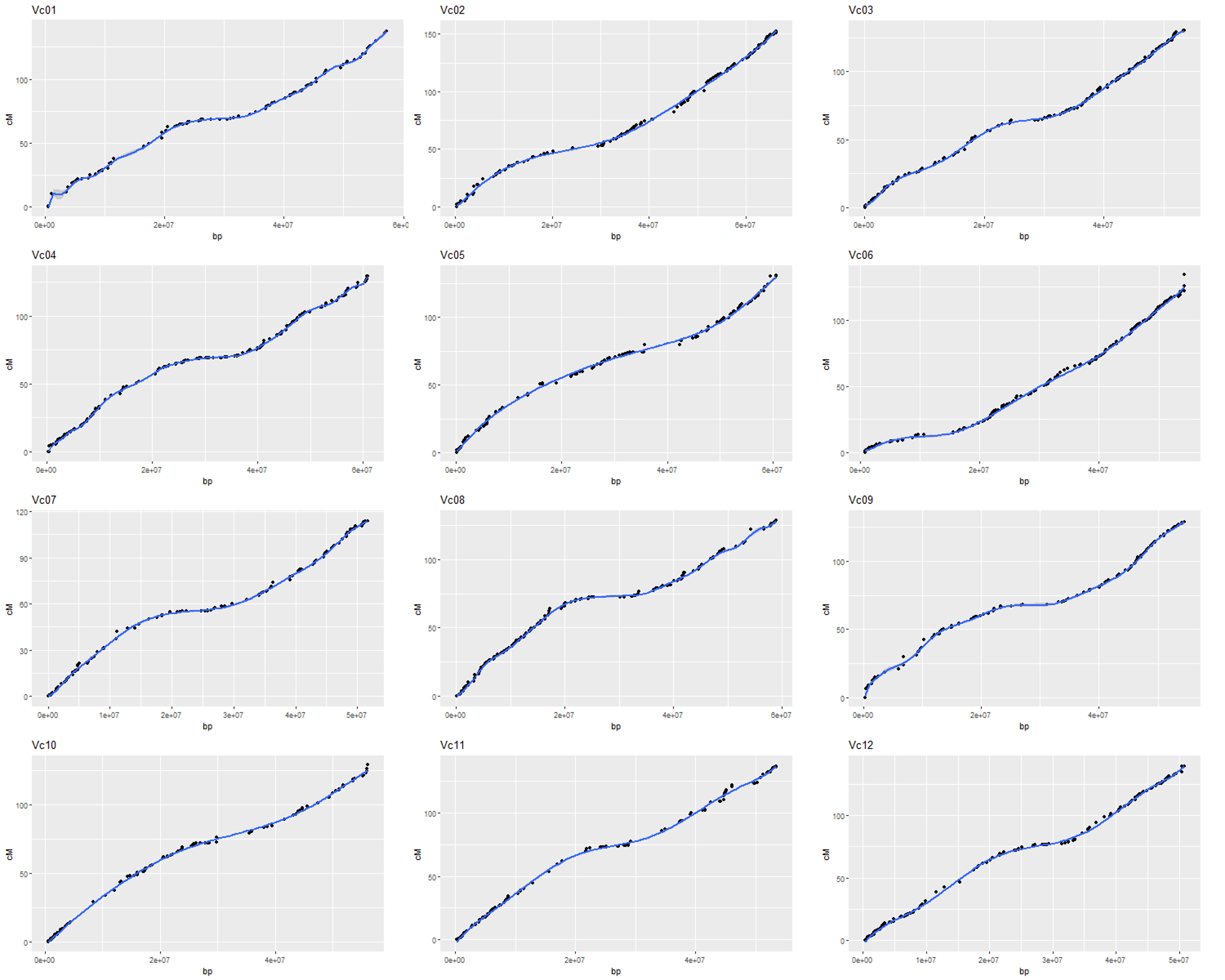

Supplement: Supplementary file 1 [file plants-12-01346-s001.zip › Figure_S2.png]

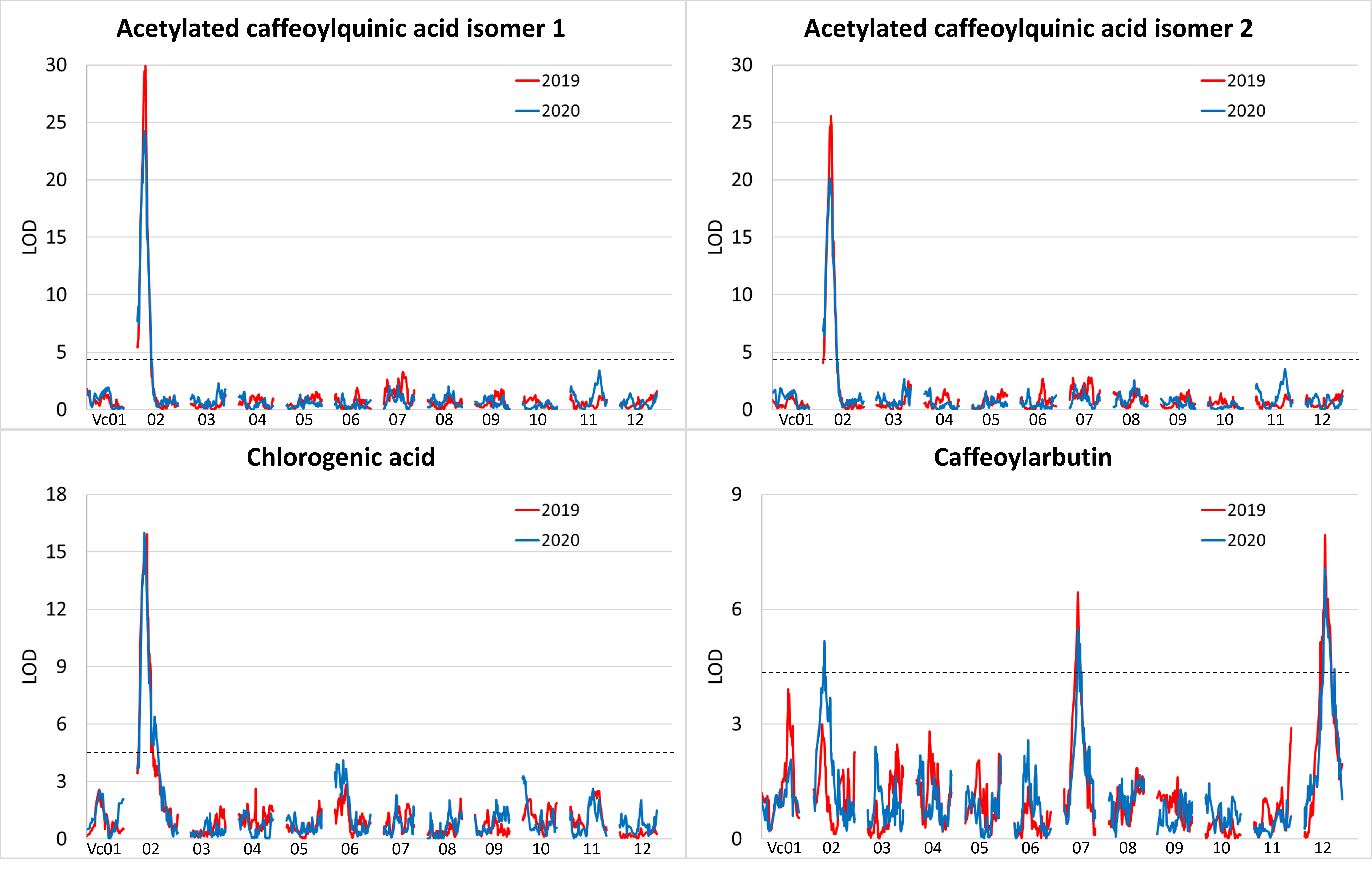

Supplement: Supplementary file 1 [file plants-12-01346-s001.zip › Figure_S3.png]

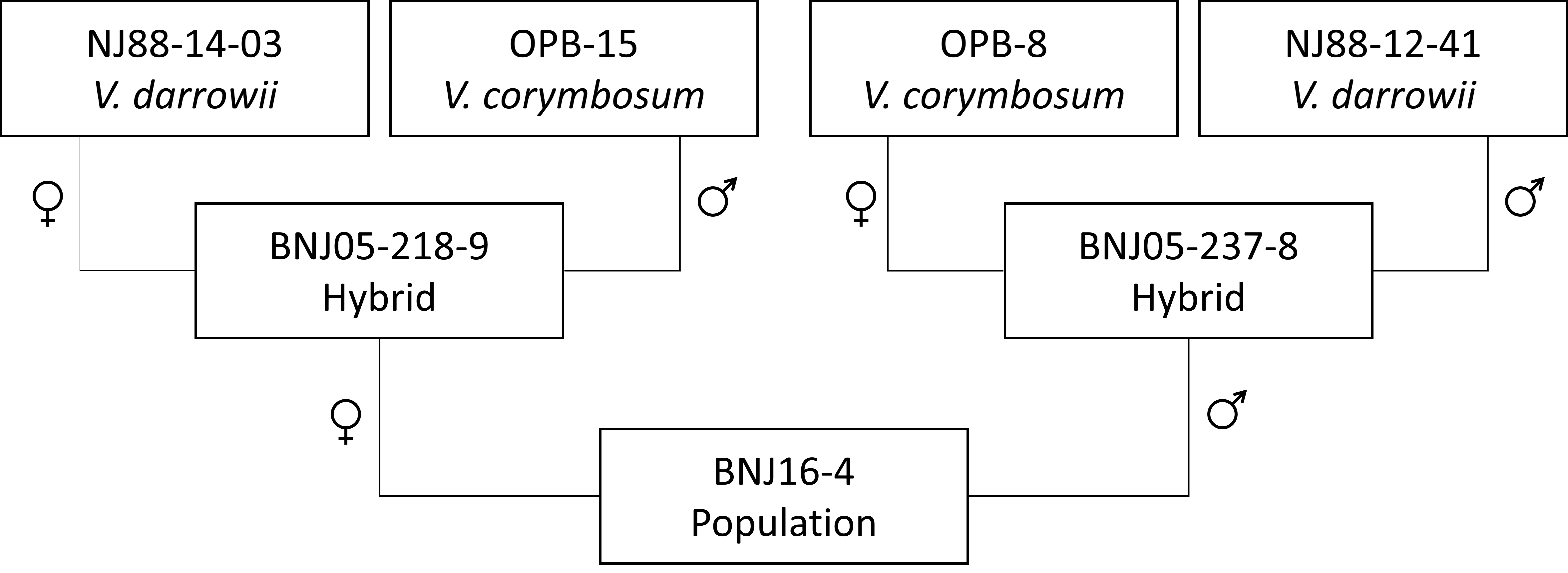

Supplement: Supplementary file 1 [file plants-12-01346-s001.zip › Figure_S4.png]
